# Targeting tumorigenesis: development and use of mTOR inhibitors in cancer therapy

**DOI:** 10.1186/1756-8722-2-45

**Published:** 2009-10-27

**Authors:** RuiRong Yuan, Andrea Kay, William J Berg, David Lebwohl

**Affiliations:** 1 Novartis Oncology, Florham Park, NJ, USA

## Abstract

The mammalian target of rapamycin (mTOR) is an intracellular serine/threonine protein kinase positioned at a central point in a variety of cellular signaling cascades. The established involvement of mTOR activity in the cellular processes that contribute to the development and progression of cancer has identified mTOR as a major link in tumorigenesis. Consequently, inhibitors of mTOR, including temsirolimus, everolimus, and ridaforolimus (formerly deforolimus) have been developed and assessed for their safety and efficacy in patients with cancer. Temsirolimus is an intravenously administered agent approved by the US Food and Drug Administration (FDA) and the European Medicines Agency (EMEA) for the treatment of advanced renal cell carcinoma (RCC). Everolimus is an oral agent that has recently obtained US FDA and EMEA approval for the treatment of advanced RCC after failure of treatment with sunitinib or sorafenib. Ridaforolimus is not yet approved for any indication. The use of mTOR inhibitors, either alone or in combination with other anticancer agents, has the potential to provide anticancer activity in numerous tumor types. Cancer types in which these agents are under evaluation include neuroendocrine tumors, breast cancer, leukemia, lymphoma, hepatocellular carcinoma, gastric cancer, pancreatic cancer, sarcoma, endometrial cancer, and non-small-cell lung cancer. The results of ongoing clinical trials with mTOR inhibitors, as single agents and in combination regimens, will better define their activity in cancer.

## Introduction

The mammalian target of rapamycin (mTOR) is a serine/threonine kinase that is ubiquitously expressed in mammalian cells [1]. Through its downstream effectors, 4EBP1 and P70S6 kinase (S6K), mTOR is involved in the initiation of ribosomal translation of mRNA into proteins necessary for cell growth, cell cycle progression, and cell metabolism [1]. mTOR senses and integrates signals initiated by nutrient intake, growth factors, and other cellular stimuli to regulate downstream signaling and protein synthesis. This regulation can prevent cells from responding to growth and proliferation signals when the supply of nutrients and energy within the cell is insufficient to support these cellular processes and can allow cells to respond to these signals when nutrients and energy are abundant [2]. Inappropriate mTOR activation has been implicated in the pathogenesis of numerous tumor types [3,4]. This article will describe the normal functions of mTOR, its dysregulation in cancer, and its value as a target for inhibition by anticancer agents.

### mTOR Structure and Function

mTOR is a key protein evolutionarily conserved from yeast to man; embryonic mutations in mTOR are lethal [3]. Two mTOR complexes participate in 2 functionally disparate protein complexes, mTOR complex 1 (mTORC1) and mTOR complex 2 (mTORC2). mTORC1 is associated with the activity that correlates with the cellular endpoints observed through the inhibitory effects of rapamycin. Rapamycin was known almost 20 years before its substrate, a large (250 kDa) protein, designated "target of rapamycin" (TOR), was identified. The mammalian orthologue is termed "mammalian target of rapamycin" [5]. mTORC2 is not responsive to rapamycin, and while this mTOR complex is not well defined, its function appears to be involved in cytoskeletal dynamics. For the purposes of this article, we will discuss only mTORC1 and refer to it as mTOR.

**Figure 1:**
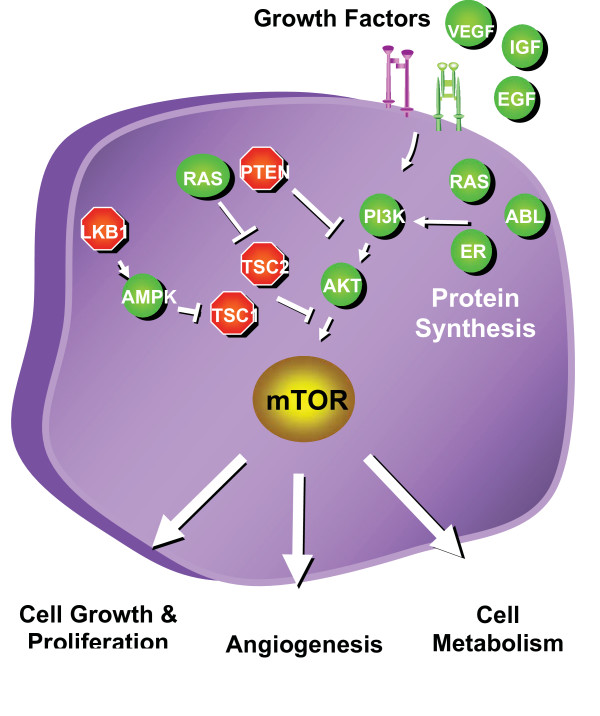
**Positive and negative regulators of mTOR activity**. Proteins that activate mTOR are shown in green, and those that suppress mTOR activity are shown in red.

In normal cells, positive and negative regulators upstream of mTOR control its activity (Figure 1) [3]. Positive regulators include growth factors and their receptors, such as insulin-like growth factor-1 (IGF-1) and its cognate receptor IFGR-1, members of the human epidermal growth factor receptor (HER) family and associated ligands, and vascular endothelial growth factor receptors (VEGFRs) and their ligands, which transmit signals to mTOR through the PI3K-Akt and Ras-Raf pathways. Negative regulators of mTOR activity include phosphatase and tensin homolog (PTEN), which inhibits signaling through the PI3K-Akt pathway, and tuberous sclerosis complex (TSC) 1 (hamartin) and TSC2 (tuberin). Phosphorylation of TSC2 by Akt releases its inhibitory effect on mTOR and upregulates mTOR activity. Another negative regulator, LKB1, is in an energy-sensing pathway upstream of TSC [6].

mTOR signals through its downstream effectors, 4EBP1 and S6K, to initiate ribosomal translation of mRNA into protein. mTOR activation leads to increased synthesis of multiple proteins, including several that have been implicated in the pathogenesis of multiple tumor types. Examples include cyclin D1, which is instrumental in allowing progression of cells through the cell cycle [7], hypoxia-inducible factors (HIFs), which drive the expression of angiogenic growth factors (eg, vascular endothelial growth factor [VEGF], platelet-derived growth factor-β [PDGFβ ]) [1], and certain proteins involved in nutrient transport [8].

### mTOR Is Implicated in the Development and Progression of Various Tumor Types

The PI3K-Akt pathway is an important regulator of cell growth and survival [9]. In many tumors, components of this pathway are dysregulated (Table 1), permitting unrestricted cancer cell growth and proliferation and evasion of apoptosis, contributing to tumorigenesis [3,4]. Increased mTOR activity appears to be promoted by dysregulation of the regulators of mTOR, in particular, the PI3K/Akt/mTOR pathway.

**Table 1:** Components of the PI3K/Akt/mTOR Pathway Frequently Deregulated in Cancer

**Target**	**Type of Protein**	**Genetic Aberration**	**Tumor Types**
EGFR [88]	Tyrosine kinase receptor	Amplification, mutation	Colorectal, lung, gastric, pancreas, liver, lung, others

HER2 [89]	Tyrosine kinase receptor	Expression	Breast

ER [90]	Hormone receptor	Expression	Breast, endometrial

PTEN [91]	Lipid phosphatase	Silencing, allele loss	Glioma, endometrial, prostate, melanoma, breast

PI3KCA [92]	Serine-threonine kinase	Mutations	Colorectal, breast, lung, brain

TSC1 [93]	TSC complex protein	Mutation	Bladder

LKB1 [94,95]	Serine-threonine kinase	Mutation, silencing	Colorectal, lung

K-ras [96]	GTP-binding kinase	Mutation	Colorectal, pancreas, lung, melanoma

BCR-ABL [97]	Tyrosine kinase	Translocation	CML, ALL

VHL [98]	Ubiquitin ligase	Loss of heterozygosity, mutation, silencing	Kidney, hemangioblastomas

mTOR signaling is critical in the development of many tumors, including renal cell carcinoma (RCC), in which mTOR can play a specific role in the angiogenesis pathways that are frequently up-regulated [10]. The pathobiology of RCC, and tumors with clear cell histology in particular, involves mutation or loss of expression of the von Hippel-Lindau (VHL) gene. In about 75% of clear cell RCC cases, the function of the VHL protein is lost. VHL is a ubiquitin ligase that targets HIF-1α for proteasomal degradation, and its loss results in the accumulation of HIF [11]. mTOR regulates the synthesis of HIF-1α, and when loss of VHL function coincides with upregulation of mTOR activity, this scenario can drive overexpression of angiogenic growth factors, including VEGF and PDGFβ [11]. Proteins in the PI3K/Akt/mTOR pathway that are dysregulated in cancer, such as PTEN, IGF-1/IGF-1R, and TSC, also contribute to RCC tumorigenesis (Table 2). Hereditary loss of TSC is associated with an increased incidence of several tumor types, including kidney tumors [12].

**Table 2:** Components of the PI3K-Akt-mTOR Pathway Deregulated in RCC

**Target**	**Type of Protein**	**Genetic Aberration**	**Potential Relevance in RCC**
IGF-1, IGF-1R [99]	Growth factor, tyrosine kinase receptor	Overexpression	Patients with IGF-1R+ clear cell RCC (ccRCC) have shorter survival than those with IGF-1R-negative ccRCC [100]

PTEN [91]	Lipid phosphatase	Silencing, allele loss	PTEN expression may be lost early in RCC carcinogenesis [101] PTEN-deficient tumor cells have increased sensitivity to mTOR inhibition [102]

TSC1/TSC2 [12]	TSC complex protein	Hereditary loss	Hereditary loss leads to an increased incidence of several tumor types, including kidney tumors [12]. The TSC tumor suppressors are key components in the upstream regulation of mTOR [103].

VHL [98]	Ubiquitin ligase	Loss of heterozygosity, mutation, silencing	Up to 75% of clear cell RCCs have lost the function of the von Hippel-Lindau (*VHL*) gene [11], resulting in accumulation of HIF-1α, a protein that controls the expression of genes involved in angiogenesis.

This defined role for mTOR activity in the cellular processes that contribute to the development and progression of multiple tumor types has established mTOR as a major link in tumorigenesis. Preclinical data have supported the pivotal role of mTOR in cancer and led to the development of mTOR inhibitors as a therapeutic target [13].

### The Development of mTOR Inhibitors

Rapamycin (sirolimus), an antifungal agent with immunosuppressive properties, was isolated in 1975 on the island of Rapa Nui [14]. In the 1990s, the substrate for rapamycin was identified as TOR, the mammalian analogue is designated mTOR [4]. Rapamycin was analyzed for anticancer activity against a panel of human cancer cell lines by the US National Cancer Institute in the 1980s and was found to have broad anticancer activity [15]. However, clinical development of mTOR inhibitors as anticancer agents was less than successful at that time due to unfavorable pharmacokinetic properties [13]. In the interim, sirolimus (Rapamune, Wyeth Pharmaceuticals) has been used in combination with corticosteroids and cyclosporine as a preventive therapy for kidney transplant rejection in the United States and Europe [16]. Additionally, an orally available rapamycin analogue, everolimus, is approved for use as a preventive therapy for transplant rejection in renal and cardiac transplantation patients in Europe [17-19].

**Figure 2:**
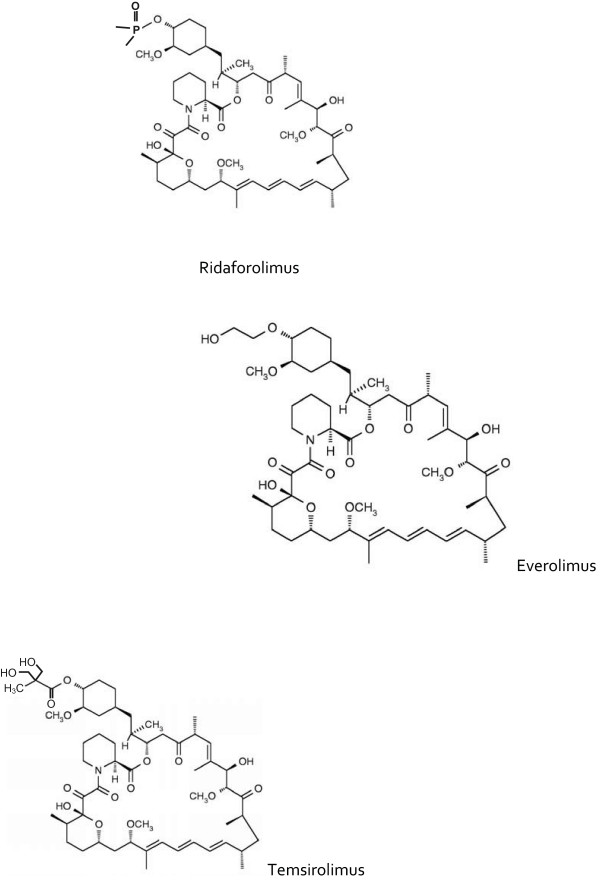
**Chemical structures of ridaforolimus, everolimus, and temsirolimus**.

The revival of mTOR inhibitor evaluation as anticancer agents began with rapamycin analogues that have a more favorable pharmacokinetic profile than the parent molecule. Currently, those analogues include temsirolimus (CCI-779, Torisel, Wyeth Pharmaceuticals), everolimus (RAD001, Afinitor, Novartis Pharmaceuticals), and ridaforolimus (AP23573; formerly deforolimus, ARIAD Pharmaceuticals). The chemical structures of these compounds are shown in Figure 2. These agents have a similar mechanism of action, though they have disparate pharmacokinetic properties.

These drugs are small molecule inhibitors that function intracellularly, forming a complex with the FK506 binding protein-12 (FKBP-12), which is then recognized by mTOR. The resultant complex prevents mTOR activity [4]. These inhibitors are similar to rapamycin in that they affect only mTORC1, but not mTORC2. The function of mTORC2 and its role in normal and cancerous cells remains relatively undefined. mTOR inhibition results in the abrogation of a number of cellular endpoints implicated in tumorigenesis. Many of the key acquired capabilities of cancer cells can be affected by the inhibition of dysregulated mTOR activity, including cell cycle progression, cellular metabolism, cellular survival, and angiogenesis [3,13].

Differences among the mTOR inhibitors include metabolism, formulation, and schedule of administration. Temsirolimus is a pro-drug, and its primary active metabolite is rapamycin (sirolimus) [20]. Temsirolimus is approved by the US Food and Drug Administration (FDA) and the European Medicines Agency (EMEA) for the treatment of advanced RCC. It is administered intravenously on a once-weekly schedule. It is supplied in vials that must be refrigerated and protected from light, and it must be diluted twice before administration [21]. Ridaforolimus is not a pro-drug [22], but like temsirolimus, it is also administered intravenously on an intermittent schedule, although an oral formulation is currently being evaluated in sarcoma [23,24]. Everolimus is an orally available mTOR inhibitor that is typically administered on a continuous daily schedule. Everolimus is also being administered in clinical trials on a weekly schedule, but the continuous, daily dosing schedule appears to be optimal for certain tumor types [25]. Weekly administration is being investigated in combination regimens. Everolimus has recently obtained US FDA and EMEA approval for the treatment of advanced RCC after failure of treatment with sunitinib or sorafenib.

### Phase I Studies and Safety of mTOR Inhibitors

The phase I dose-finding studies for temsirolimus and ridaforolimus were conventional in design, in that they attempted to establish a maximum tolerated dose through dose escalation [22,26,27]. In contrast, the everolimus studies relied on pharmacokinetic and pharmacodynamic modeling, as well as traditional dose-escalation methodology, to provide for rational selection of the optimal doses and schedules for exploration in future clinical trials [25,28,29]. Data from these studies showed that mTOR inhibition with everolimus was dose dependent and that continuous daily dosing produced more profound mTOR inhibition than weekly dosing, [25,28,29] and everolimus had acceptable tolerability at the highest dosages studied [25,29]. The results of phase I studies conducted with ridaforolimus, everolimus, and temsirolimus are summarized in Table 3[22,25,26,29].

**Table 3:** mTOR Inhibitors: Phase I and Pharmacokinetic Data

**mTOR Inhibitor**	**T_1/2 _(h)**	**Primary Metabolite**	**Dose and Schedule**	**MTD**	**DLTs (all grade 3)**	**Suggested Phase II Dose**
Ridaforolimus [22]	56-74	Not sirolimus pro-drug	3-28 mg/d IV × 5 d q 2 wk	18.75 mg	Mouth sores	12.5 mg IV × 5 d q 2 wk

Everolimus [25,29]	~30	Not sirolimus pro-drug	Oral daily: 5-10 mg/d Oral weekly: 5-70 mg/wk	NR	Daily: hyperglycemia, stomatitis Weekly: stomatitis, fatigue, neutropenia, hyperglycemia	Daily: 10 mg Weekly: 50-70 mg

Temsirolimus [26]	13-22	Sirolimus	7.5-220 mg/m^2^/wk	Formal definition of MTD not met	Neutropenia, thrombocytopenia, hypophosphatemia; asthenia, diarrhea; manic-depressive syndrome, stomatitis; ALT elevation	25, 75, and 250 mg (flat dose) wkly

*In heavily pretreated patients.

^†^In minimally pretreated patients, no MTD was established, but the maximum acceptable dose was 19 mg/m^2 ^due to grade 3 stomatitis and dose reductions in 2 patients.

NR = Not reached.

Phase I safety analyses showed that the mTOR inhibitors are generally well tolerated. Class-specific adverse events (AEs) are consistently observed with each of the 3 agents, most commonly including mild to moderate stomatitis/oral mucositis, skin rash/erythema, and metabolic abnormalities (hyperglycemia and hyperlipidemia) [22,25,26,29]. Noninfectious pneumonitis also appears to be a class effect of mTOR inhibitors and has been reported with everolimus and temsirolimus [25,30,31]. Temsirolimus has been associated with infusion reactions, and the administration protocol was altered to include diphenhydramine pretreatment before temsirolimus infusion in subsequent studies [20].

The pivotal role that mTOR plays in cellular signaling suggests a broad range of clinical utility, and indeed, phase I clinical evaluations of all 3 mTOR inhibitors provided preliminary evidence of anticancer activity in multiple tumor types. Activity in RCC was seen with each agent. Clinical programs for each of these agents continue to develop in multiple tumor types.

### mTOR Inhibitors in Renal Cell Carcinoma

#### Temsirolimus

Based on phase I activity in RCC, a phase II temsirolimus study was conducted in 111 heavily pretreated patients with advanced RCC of all risk categories [31]. Temsirolimus was administered intravenously once weekly at fixed doses of 25 mg, 75 mg, or 250 mg. This study supported the activity of temsirolimus seen in phase I trials. One complete response (CR), 7 partial responses (PRs), and 29 minor responses were observed. Dose level did not appear to influence response, but more dose reductions and discontinuations were observed at the higher dose levels, suggesting that the 25-mg dose should be used for future studies. In addition, 5 patients treated with temsirolimus 75 mg developed pneumonitis. Retrospective classification of patients into good, intermediate, and poor risk groups similar to the Memorial Sloan-Kettering Cancer Center (MSKCC) prognostic risk criteria for previously untreated patients [32] suggested that temsirolimus was more effective in patients with intermediate and poor risk than in those with favorable risk [31].

Based on these results, a phase III double-blind randomized trial compared temsirolimus, interferon-α (IFN-α), and temsirolimus + IFN-α in 626 poor-risk (≥3 of 6 prognostic risk factors) patients with previously untreated RCC [33]. Temsirolimus was administered at a dose of 25 mg weekly. Compared with IFN-α alone, temsirolimus significantly improved overall survival (7.3 months vs. 10.9 months, p = 0.008) and reduced the risk of death by 27%. Combination therapy did not improve survival compared with IFN alone. Based on the results of this study, temsirolimus was approved for use in metastatic RCC in the United States and Europe in 2007 [16]. A subset analysis of the phase III trial showed that the benefit of temsirolimus may be primarily in the poor-risk, non-clear-cell RCC population. The common adverse events observed with temsirolimus were asthenia, stomatitis, rash, nausea, anorexia, and dyspnea. The common abnormal laboratory findings in this trial were hyperglycemia, hypercholesterolemia, and anemia. The most common grade 3/4 adverse events observed with temsirolimus (regardless of causality) in this trial included anemia (20%), hyperglycemia (11%), asthenia (11%), and dyspnea (9%). Most adverse events were manageable with supportive care or dose reduction [34]. An ongoing phase III trial is evaluating temsirolimus plus bevacizumab vs. IFN-α plus bevacizumab in patients with advanced clear cell RCC [35].

#### Everolimus

Phase II investigation of daily everolimus in 41 patients with metastatic RCC (of whom 83% had received prior systemic therapy) showed encouraging activity, with a median progression-free survival (PFS) of 11.2 months, a median overall survival of 22.1 months, and a response rate of 14%; furthermore, more than 70% of patients had a response or stable disease (SD) lasting for ≥6 months [36]. Currently, sorafenib and sunitinib are among the recommended first-line treatment agents for metastatic RCC [37]. When these VEGFR-targeted therapies are exhausted, until recently there was no evidence that demonstrated clearly which therapy should be offered next. To address this unmet need, a phase III double-blind, randomized, placebo-controlled trial (RECORD-1) was initiated to evaluate the activity of daily oral everolimus in patients whose disease had progressed following therapy with VEGFR tyrosine kinase inhibitors (TKIs) [38]. Eligibility criteria included disease progression during or within 6 months of treatment with sunitinib and/or sorafenib. Previous treatment with cytokines or bevacizumab was permitted. A total of 416 patients from 86 centers were enrolled and stratified by the number of previous treatments (sorafenib or sunitinib [1 TKI] vs. sorafenib as well as sunitinib [2 TKIs]) and MSKCC prognostic risk group (favorable, intermediate, or poor). Patients were then randomized 2:1 to treatment with everolimus (10 mg daily) and best supportive care (BSC) or to placebo and BSC. Treatment was continued until disease progression, unacceptable toxicity, death, or discontinuation for other reasons. Patients randomized to placebo and BSC were allowed to cross over to everolimus at disease progression. At baseline, the majority of patients were in the intermediate MSKCC risk group (56% and 57% in everolimus and placebo groups, respectively), and most had received only 1 prior TKI (74% in both groups). After the second interim analysis, the study was terminated early after 191 progression events were observed because the prespecified efficacy endpoint was met [38]. Based on analyses from the end of the double-blind period, everolimus significantly improved PFS vs. placebo: 4.9 months vs. 1.9 months, respectively (hazard ratio: 0.33; 95% confidence interval [CI]: 0.25-0.43; p < 0.001) [39]. Everolimus significantly increased median PFS in each MSKCC risk group and regardless of whether patients had received 1 or 2 prior TKIs. Similar to another mTOR inhibitor, temsirolimus, the most common adverse events of all grades observed in everolimus-treated patients included fatigue, stomatitis, rash, nausea, anorexia, and stomatitis. The classic mTOR inhibitor-related abnormal laboratory findings, including anemia, hypercholesterolemia, hypertriglyceridemia, and hyperglycemia were observed [38]. The most common treatment-related grade 3/4 adverse events with everolimus were lymphopenia (15%), hyperglycemia (12%), and anemia (9%). Most adverse events were manageable with supportive care or dose reduction. Noninfectious pneumonitis associated with rapamycin or rapamycin derivative treatment was previously reported [31] and also was seen with everolimus in this trial. Approximately 14% of patients receiving everolimus developed noninfectious pneumonitis; however, only 3% of patients had grade 3 severity and no patients had grade 4 severity. Most cases of noninfectious pneumonitis were mild (grade 1/2) and medically manageable [39].

Based on these clinical trial data, algorithms that define evidence-based treatment options for metastatic RCC have been developed to include mTOR inhibitors, including temsirolimus for the treatment of patients with metastatic RCC with selected risk features and everolimus for the treatment of metastatic RRC in patients whose disease recurred following prior TKI therapy [40,41].

#### Ongoing Trials in RCC

Further development of mTOR inhibitors for the treatment of RCC is ongoing in combination with antiangiogenic agents such as bevacizumab, sorafenib, and sunitinib. The combination of everolimus and bevacizumab is active and well tolerated in patients with metastatic clear cell RCC; cohorts of first-line and previously treated patients were examined in the study [42]. A randomized trial (RECORD-2) is ongoing to evaluate everolimus plus bevacizumab vs. interferon-α plus bevacizumab in patients with progressive, metastatic clear cell RCC [43]. A planned randomized trial (RECORD-3) will compare first-line everolimus followed by second-line sunitinib vs. the alternate sequence in patients with metastatic RCC [44].

### Future Directions With mTOR Inhibitors

The results of preclinical and phase I studies, as well as data from biomarker studies showing oncogenic transformation in mTOR-linked pathways (Table 1) suggest that mTOR inhibitors may have anticancer activity in many tumor types. In addition to RCC, pivotal clinical trials with mTOR inhibitors are ongoing in many cancers, including but not limited to: neuroendocrine tumors (NET), pancreatic islet cell tumors, breast cancer, diffuse large B-cell lymphoma, hepatocellular carcinoma, and gastric cancer. Phase II studies have also been performed in pancreatic adenocarcinoma, sarcoma, endometrial cancer, and non-small cell lung cancer (NSCLC).

#### Neuroendocrine Tumors

Neuroendocrine tumors are characterized by their ability to manufacture and secrete peptides that cause hormonal syndromes [45]. Although these tumor types are rare, their incidence appears to be increasing. Metastatic low-grade NETs are generally resistant to chemotherapy and are relatively incurable, though hormonal symptoms are managed with somatostatin analogues [46,47].

Temsirolimus and everolimus have both been studied in patients with advanced NET. Weekly infusions of temsirolimus demonstrated modest activity in patients (n = 37) with progressive NET in a phase II study, with an overall response rate (ORR) of 5.6% [30]. In another phase II study, daily administration of everolimus in combination with monthly intravenous octreotide (a somatostatin analogue) for up to 12 months provided more notable results in one cohort of patients (n = 30) with carcinoid or islet cell tumors, with an ORR of 20%, a median PFS duration of 60 weeks, and acceptable tolerability [48].

The RADIANT-1 phase II trial evaluated everolimus in patients with metastatic pancreatic NETs whose disease progressed on prior cytotoxic chemotherapy [49]. Patients were enrolled into 2 strata based on whether they were previously receiving octreotide LAR therapy; patients in stratum 1 received oral everolimus 10 mg/day alone (n = 115) and patients in stratum 2 received oral everolimus 10 mg/day plus octreotide LAR intramuscularly every 28 days at their current dose (n = 45). Most patients had been diagnosed > 2 years before study entry, and over 90% of patients in both strata had liver metastases. The ORR (by central radiology) was 9.6% in stratum 1 and 4.4% in stratum 2. Stable disease was maintained in 68% of patients in stratum 1 and 80% of patients in stratum 2. Median PFS (by central radiology) was 9.7 months in stratum 1 and 16.7 months in stratum 2, and median overall survival was 24.9 months in stratum 1 and not reached in stratum 2. Treatment was generally well tolerated in both strata. Based on these encouraging results, 2 subsequent RADIANT studies are ongoing. RADIANT-2 is a randomized, double-blind, placebo-controlled, multicenter phase III study of octreotide LAR with everolimus or placebo in patients with advanced carcinoid tumors [50]. The RADIANT-2 study has completed accrual. RADIANT-3, a randomized, double-blind phase III trial, has completed enrollment and is currently ongoing to further evaluate everolimus in the treatment of patients with pancreatic NET [51].

#### Breast Cancer

In breast cancer, resistance to treatment with endocrine therapies and HER-2 targeted agents inevitably develops in many patients [52,53]. mTOR inhibitors have shown clinical activity in patients with advanced breast cancer [54,55] and are being actively investigated in this setting in combination with other agents that have shown clinical activity in metastatic breast cancer (MBC).

A phase III study of temsirolimus in combination with letrozole did not demonstrate benefit over letrozole alone in patients with MBC and was terminated at an interim analysis [56]. These results may reflect the need for better biomarker-based patient selection.

A study evaluating 2 schedules of oral everolimus administration (continuous daily vs. weekly) in patients with MBC showed that continuous daily administration produced greater tumor shrinkage [55]. In 2006, a phase III trial evaluating temsirolimus in combination with endocrine therapy (letrozole) in estrogen receptor-positive (ER+) women with advanced breast cancer was discontinued due to missed endpoints involving efficacy [56,57]. The development of mTOR inhibitors in MBC continued, and investigators approached a phase II everolimus neoadjuvant trial by first attempting to identify biomarkers to predict which patients might be more likely to respond to a combination including an mTOR inhibitor and endocrine therapy. In this study, the response rate by clinical palpation in patients treated with everolimus and letrozole was superior to that in patients treated with letrozole alone [58]. Inhibition of tumor proliferation, as reflected by decreased Ki67-positive tumor cells, was more prominent with everolimus plus letrozole compared with letrozole alone (mean reduction at day 15 relative to baseline 90.7% in everolimus group vs. 74.8% in placebo group; p = 0.0002), and inhibition of mTOR activity (decreased pS6K levels) was observed in patients treated with the combination [58]. Results of a phase I trial of this combination in patients with MBC whose disease was stable or had progressed after 4 months with letrozole alone showed that it was well tolerated and active in this patient population [59]. An ongoing randomized, double-blind, placebo-controlled phase III trial (BOLERO-2) is evaluating everolimus in combination with exemestane in patients with estrogen-receptor positive locally advanced or metastatic breast cancer who are refractory to letrozole or anastrozole [60].

Ongoing phase I studies are evaluating the addition of everolimus to cytotoxic chemotherapy and HER2-targeted therapy in hopes that these combinations can delay or overcome trastuzumab resistance in HER2-positive breast cancer [61,62]. Either daily or weekly everolimus was administered in combination with weekly chemotherapy and trastuzumab. Preliminary results have been encouraging: an unexpected degree of anticancer activity has been seen in patients resistant to both taxanes and trastuzumab, and the combinations with everolimus were well tolerated. A randomized, double-blind, placebo-controlled phase III trial (BOLERO-1) is planned to evaluate the addition of everolimus to paclitaxel and trastuzumab as first-line therapy in patients with HER2-positive locally advanced or metastatic breast cancer [63].

#### Lymphoma

Lymphomas appear to be sensitive to mTOR inhibitor therapy. Everolimus (10 mg/day PO, 28-day/cycle until progression or toxicity) was evaluated in 145 previously treated patients with aggressive lymphomas or uncommon lymphomas, including 77 with aggressive NHL, 41 with indolent NHL, 8 with T-cell NHL, and 17 with Hodgkin disease [64]. Patients had received a median of 4 prior therapies. The ORR was 33% (48/145), with 5 patients achieving CR and 43 patients achieving PR. The median time to progression in all patients was 4.3 months. Everolimus was generally well tolerated, and grade 3/4 adverse events included anemia (16%), neutropenia (17%), thrombocytopenia (35%), hypercholesterolemia (1%), hyperglycemia (5%), and hypertriglyceridemia (n = 1). In the 17 patients with Hodgkin lymphoma, 15 patients were evaluable for response; 7 (47%) had PRs [65]. An open-label phase II trial (PILLAR-1) is ongoing to evaluate everolimus in previously treated patients with mantle cell lymphoma (MCL) who are refractory or intolerant to bortezomib therapy [66]. An ongoing randomized, double-blind, multicenter phase III study (PILLAR-2) is evaluating everolimus as adjuvant therapy in poor-risk patients with diffuse large B cell lymphoma who achieved complete remission with first-line rituximab and chemotherapy [67].

Temsirolimus, administered intravenously at 25 mg weekly, also has shown activity in NHL subtypes. Response rates of 36% (DLCL) and 56% (follicular lymphoma) were observed in a 56-patient study [68]. In relapsed MCL, 1 CR and several PRs were observed in a phase II temsirolimus study [69]. Positive results were also recently reported from a large open-label phase III study, which compared temsirolimus, 175 mg three times a week followed by either 75 mg or 25 mg weekly, with investigator's choice of therapy in 162 patients with relapsed or refractory MCL [70]. The ORR was significantly higher in the temsirolimus 175 mg/75 mg dose group (22%) vs. investigator's choice (2%; p = 0.0019). Median PFS was 4.8 months with temsirolimus 175 mg/75 mg vs. 3.4 months with temsirolimus 175 mg/25 mg and 1.9 months with investigator's choice of therapy (p = 0.0009 for temsirolimus 175 mg/75 mg vs. investigator's choice). No significant differences in OS were observed (12.8 months vs. 10.0 months vs. 9.7 months with temsirolimus 175 mg/75 mg, temsirolimus 175 mg/25 mg, and investigator's choice, respectively). The most common grade 3/4 adverse events observed in the 2 temsirolimus treatment groups were thrombocytopenia (52%-59%), anemia (11%-20%), asthenia (13%-19%), and diarrhea (7%-11%). Recently, the EMEA Committee for Medicinal Products for Human Use rendered a positive opinion for temsirolimus to be approved in Europe to treat patients with relapsed/refractory MCL [71].

Ridaforolimus was evaluated in a phase II trial of 52 heavily pretreated patients with a variety of hematologic malignancies, including acute myelogenous leukemia (AML), chronic myelogenous leukemia (CML), myelodysplastic syndrome (MDS), acute lymphocytic leukemia (ALL), chronic lymphocytic leukemia (CLL), agnogenic myeloid metaplasia (AMM), and MCL. The PR rate was 10% (2 of 7 patients with AMM and 3 of 9 patients with MCL), and SD/hematologic improvement occurred in 40% (4 of 22 patients with AML, 1 of 2 patients with MDS, 3 of 7 patients with AMM, 6 of 8 patients with CLL, 2 of 2 patients with T-cell lymphoma, and 4 of 9 patients with MCL). Ridaforolimus was well tolerated [72].

Overall, these encouraging results provide support for conducting additional clinical trials with mTOR inhibitors in both NHL and Hodgkin lymphoma.

#### Gastric Cancer

Everolimus was evaluated in a multicenter phase II study involving previously treated patients with metastatic gastric cancer [73]. In an analysis of trial data after 54 patients were enrolled, the disease control rate (proportion of patients with CR, PR, or SD as the best overall response at the objective tumor assessment performed according to RECIST) was 55%, median PFS was 2.7 months, and tolerability was acceptable [74]. These findings support the further evaluation of everolimus in patients with advanced gastric cancer. A randomized, double-blind, multicenter phase III study (GRANITE-1) is planned to compared everolimus plus BSC vs. placebo plus BSC in patients with advanced gastric cancer who progressed after 1 or 2 prior chemotherapy regimens [75].

#### Sarcoma

Of the mTOR inhibitors, ridaforolimus has been most thoroughly investigated in sarcoma. A phase II trial of temsirolimus in 41 patients failed to meet endpoints in soft tissue sarcomas [76]. In contrast, a clinical benefit response (CBR = CR + PR + SD) rate of 29% was reported in a trial of 212 patients with advanced bone and soft tissue sarcomas treated with ridaforolimus. The subset of patients who achieved a CBR had a longer median overall survival than the entire study population [77]. Results of a recent study of specimens from patients with high-grade sarcomas suggested that the level of expression of phosphorylated S6 was predictive of tumor response to ridaforolimus [78]. An oral formulation of ridaforolimus will be studied as maintenance therapy in a phase III trial, the Sarcoma Multi-Center Clinical Evaluation of the Efficacy of Ridaforolimus (SUCCEED) trial, which is currently enrolling patients with metastatic soft-tissue or bone sarcomas [79].

#### Endometrial Cancer

Clinical trials with each of the mTOR inhibitors have been conducted in endometrial cancer, and preliminary results suggest activity. Oza *et al. *reported an ORR of 26% temsirolimus in previously untreated patients with metastatic or recurrent (after hormonal therapy) endometrial cancer [80]. Trials with ridaforolimus and everolimus have been conducted in previously treated patients, and both mTOR inhibitors appear to have activity in this setting. A CBR of 33%, with 2 partial responses, was observed in patients who received ridaforolimus [81]. Similar results were observed in patients treated with daily everolimus; CBR was observed in 43% of evaluable patients [82].

#### Non-Small Cell Lung Cancer

Everolimus monotherapy was evaluated in a phase II trial involving patients with stage IIIB/IV NSCLC who had previously received = 2 prior chemotherapy regimens [83]. Patients were enrolled into 2 strata; stratum 1: prior platinum-based chemotherapy (n = 42) and stratum 2: prior chemotherapy plus prior TKI therapy (n = 43). The ORR was 4.7% (7.1% in stratum 1 and 2.3% in stratum 2), with an overall disease control rate of 47.1%. Median PFS was 2.6 months in stratum 1 and 2.7 months in stratum 2. These results prompted further investigation of everolimus in NSCLC.

The combination of everolimus with the EGFR tyrosine kinase inhibitor gefitinib was evaluated in a phase I trial of 10 patients with progressive NSCLC, based on the hypothesis that inhibition of the PI3K/Akt/mTOR pathway by both agents would result in additive or synergistic activity. Daily doses of everolimus 5 mg and 10 mg were assessed; the 10-mg dose was discontinued due to dose-limiting toxicities of grade 5 hypotension and grade 3 stomatitis. However, partial radiographic responses were found in 2 patients who received the 5-mg dose, which was tolerable in combination with gefitinib [84]. These results prompted further study of everolimus/gefitinib in a phase II trial that enrolled patients with stage IIIB/IV NSCLC who were smokers in to 2 cohorts: cohort 1 included previously untreated patients and cohort 2 included patients who had received prior platinum/docetaxel therapy [85]. In a report of results from 25 patients (11 in cohort 1 and 14 in cohort 2) a PR rate of 17% was observed.

A number of phase I and II trials evaluating everolimus in combination with TKIs and other agents are ongoing, including a phase II trial of the combination of everolimus and the EGFR tyrosine kinase inhibitor erlotinib in pretreated patients with advanced NSCLC [86] and a phase I/II trial evaluating everolimus plus carboplatin/paclitaxel and bevacizumab as first line therapy in stage IIIB/IV NSCLC [87].

## Conclusion

The improved understanding of molecular biology permits the development of agents that target dysregulated pathways in cancer cells. mTOR is a central regulator of cell growth, cell proliferation, and angiogenesis. Because mTOR is activated through cellular pathways that are dysregulated in many different types of cancer, single-agent use of mTOR inhibitors could potentially result in anticancer activity in numerous tumor types. Additionally, because mTOR is pivotal in the cellular processes that tumor cells depend on for cellular metabolism, proliferation, survival and progression, combining an mTOR inhibitor with other anticancer agents could serve to sensitize tumor cells to these agents. The potential exists for these combinations to produce additional activity or perhaps delay or prevent the development of resistance to these agents. The results of ongoing clinical trials with mTOR inhibitors, as single agents and in combination, will better define their activity in cancer.

## Competing interests

RY, AK, WJB, and DL are employed at Novartis Oncology, and all own Novartis stock.

## Authors' contributions

All authors participated in developing the concept and construct of this review and provided guidance throughout manuscript development. All authors read and approved the final manuscript.
